# Toxic Effect of Blood Feeding in Male Mosquitoes

**DOI:** 10.3389/fphys.2016.00004

**Published:** 2016-01-26

**Authors:** Mahmood R. Nikbakhtzadeh, Garrison K. Buss, Walter S. Leal

**Affiliations:** Department of Molecular and Cellular Biology, University of California-DavisDavis, CA, USA

**Keywords:** *Culex quinquefasciatus*, male blood feeding, male survival on blood, male fitness, blood meal determination

## Abstract

Blood- and sugar feeding of female mosquitoes has been frequently observed in the laboratory and in the field, but only sugar feeding of males has been reported. Here, we describe for the first time that *Culex quinquefasciatus* males feed on blood as well. Blood feeding easily happened on a blood-soaked cotton roll and, to a lesser extent, through a thin artificial layer. Mating history of a male specimen does not affect his blood feeding behavior. Male mosquitoes feed on blood even when they have a readily available sugar source. Nevertheless, feeding on blood reduces the survival rate of males to just a few days, as compared to more than a month for mosquitoes fed only on sugar. Comparing survival of male mosquitoes fed on blood only, sugar only, and a combination of both clearly demonstrated that mortality is not affected by malnutrition (reduced sugar levels), but rather due to ingested blood. On average male mosquitoes ingested ca. 0.5 μl of blood, i.e., about 10% of the amount of blood ingested by an engorged female. Although this unexpected observation of blood feeding in the laboratory by male mosquitoes is interesting, structural impairment prevents male feeding on vertebrate blood. In agreement with the literature, male and female proboscises and stylets were in general of similar size, but male mandibles were significantly shorter than female counterparts, thus explaining their inability to pierce through skin layers.

## Introduction

Mosquitoes are among the most notorious vectors of human diseases as females feed on vertebrate blood for survival and reproduction and unwittingly transmit the protozoan parasites causing malaria and many viruses that cause infections, such as dengue, yellow fever, chikungunya, and encephalitis (Leal, 2014). They destroy more lives on a year basis than war, terrorism, gun violence, and other maladies combined (Leal, 2014). Malaria alone is responsible for 627,000 cases of death annually in the world (Anonymous, 2014). Males are accomplices in this warfare by helping making more mosquitoes, but all known mosquito-borne pathogens are harbored and amplified in female mosquitoes. In general, females of most mosquito vectors need at least one blood meal before they can lay fertilized eggs, and this trait in turn enables them to transfer viral or protozoan infection to their vertebrate hosts (Kelly and Edman, 1992). In nature, females feed on both blood and sugar depending on their availability (Foster, 1995). A sugar meal provides females with enough energy to serve their physiological needs, i.e., it sustains females until they find their hosts and allows an infected mosquito to live long enough to oviposit, bite repeatedly, and to become infective (Van Handel, 1984). However, in the absence of a blood meal, mated females may lack the protein needed to synthesize yolk and develop eggs (Klowden, 1995).

It is well-known that male mosquitoes acquire their required energy from natural sources of sugar, i.e., plant nectar, honeydew, extrafloral nectaries, and rotten fruits (Van Handel, 1984; Foster, 1995) and that males do not feed on blood (Matheson, 1944; Van Handel, 1984; Ribeiro, 1987; Grant et al., 1995; Klowden, 1995; Ribeiro et al., 2001; Wahid et al., 2003). We serendipitously observed that males of our *Cx. quinquefasciatus* colony do feed on blood. Then, we designed a series of experiments to systematically examine this hitherto unknown feeding behavior. Here, we report that (i) male mosquitoes feed on blood impregnated on cotton plugs and on blood pools covered with a thin membrane even when given a choice of a sugar meal, (ii) mating history had no effect on male blood feeding, and (iii) feeding on blood was lethal to males.

## Materials and methods

### Insects

*Cx. quinquefasciatus* mosquitoes used in this study originated from a stock laboratory colony, which in turn started from adult mosquitoes collected in Merced, CA in the 1950s and is maintained by Dr. Anthon Cornel in the Kearney Agricultural Center, University of California. In Davis, mosquitoes were maintained in an Environmental Walk-in Room (Darwin Chambers Company, St. Louis, MO, USA) at 27 ± 1°C, under a photoperiod of 12:12 h (L:D) and relative humidity (RH) of 75% for the last 5 years. Adults were maintained in 30 × 30 × 30 cm aluminum collapsible cages (BioQuip, Rancho Dominguez, CA, USA), covered by dark green, UV-resistant polyester netting (24 × 20 mesh), and had continuous access to water and 10% (w/v) sucrose-soaked cotton rolls. The clock times of light-dark and dark-light transitions were at 6:45 p.m. and 6:45 a.m. (local time zone), respectively. Each cage received defibrinated sheep blood on two consecutive days using an artificial feeding apparatus. Blood was provided by the University of California, Davis, Biological Media Services, Cat # 4024. An ovicup was placed in each cage 4 days after the second blood meal. Eggs were laid in half-filled water dishes (110 ml polypropylene containers, Falcon 354014) and hatched a day later. Groups of 175 first-instar larvae were transferred to clear plastic pans (32 × 17.5 × 11 cm) and fed with the powdered Tetramin® fish food until pupation. Larvae received 70 mg of food in the first 5 days, followed by 105 mg in the following days. Adults used in our experiments emerged from plastic cups (11 cm diameter, 236.5 ml) of 650–750 pupae, held in the aforementioned BioQuip cages.

### Male blood feeding

Four-day-old male individuals of the same cohort, who had continuous access to water and sugar solution were used in assays of this study. Mosquitoes were picked with a mouth aspirator and released in a clear plastic cup (900 ml, 11 cm diameter on top), which had two screened slots (5 × 5 cm) on either side. Mosquitoes fed through a 1.5 cm opening, i.e., approximately the same diameter of a test tube, on the cup's lid. Test tubes filled with water, sugar, or blood were blocked at the open end with cotton rolls, and were inserted into the lid's opening until 3 cm of the test tube protruded inside the plastic cup (Figure S1). To provide virgin males, i.e., mosquitoes that never met mosquitoes of the opposite sex, pupae were collected and individually placed in test tubes. After emergence, males were transferred to cups, whereas mated males were collected from our rearing cages soon after mating. All laboratory assays were performed under fluorescent lighting at 27°C, 40% RH, during daytime (10:30 a.m. to 2:30 p.m.), and mosquitoes were acclimated to the lab conditions at least 2 h before onset of any assay.

### Measuring male mosquito survival on a sugar diet

We examined male mosquito survival on water (negative control), 1, 5, and 10% sucrose solutions. Mortality of each cup was recorded daily and a life table was accordingly arranged. Cups were cleaned daily, but sugar solutions and cotton rolls were replaced every other day.

### Effect of mating on blood feeding of males

Working with virgin males and females in a clear plastic cup, we observed male blood feeding for the first time. Therefore, one initial question was whether blood feeding in males is somehow connected to their mating background. In order to address this question, an experiment was designed to measure male blood feeding in mated and unmated male mosquitoes. Mated males with access to only sugar or blood were compared to unmated males, with similar access to sugar and blood. Six male specimens were released in each cup and mortality was recorded for 10 days for four replicates (*n* = 4). All males used in this experiment had already been proven to feed on a dyed sucrose solution for the first 4 days after their emergence. Picking sugar-fed males was easily possible by observing the green dye in their midgut. This way we were ensured that the males used had already fed on sugar and did not suffer from starvation before the experiment began. Blood, sugar, test tubes, and cotton rolls were replaced every other day, and data were collected every day. Before setting up any further experiments, the non-toxic food dye (McCormick & Co., Inc. Hunt Valley, MD, USA) added to the sugar solution was tested vs. the same solution with no dye in order to find out if there was any effect of such a dye on male survival. This dye contains water, propylene glycol, FD&C yellow 5 and blue 1, propylparaben. To assess the effect of the dye, 10 male specimens were released into two plastic cups, one with pure sugar solution, and the other one with a dyed sugar solution at the same concentration, and the mean of survival for each group was calculated after 2 weeks (*n* = 4).

### Measuring effect of blood meal on survival of male mosquitoes

To determine a possible negative effect of blood meal on male mosquito survival, we prepared six plastic cups, each containing 10 male specimens. Cups were supplied with water (negative control); blood (negative control); 10% sucrose, (positive control, S10%); 10% sugar, 10% blood (S10%B10%); 10% sugar, 20% blood (S10%B20%), and 10% Sugar, 40% blood (S10%B40%). In all blood/sugar mixture groups, concentration of sugar was kept constant at 10%.

### Dual choice feeding assays

**Figure 1:**
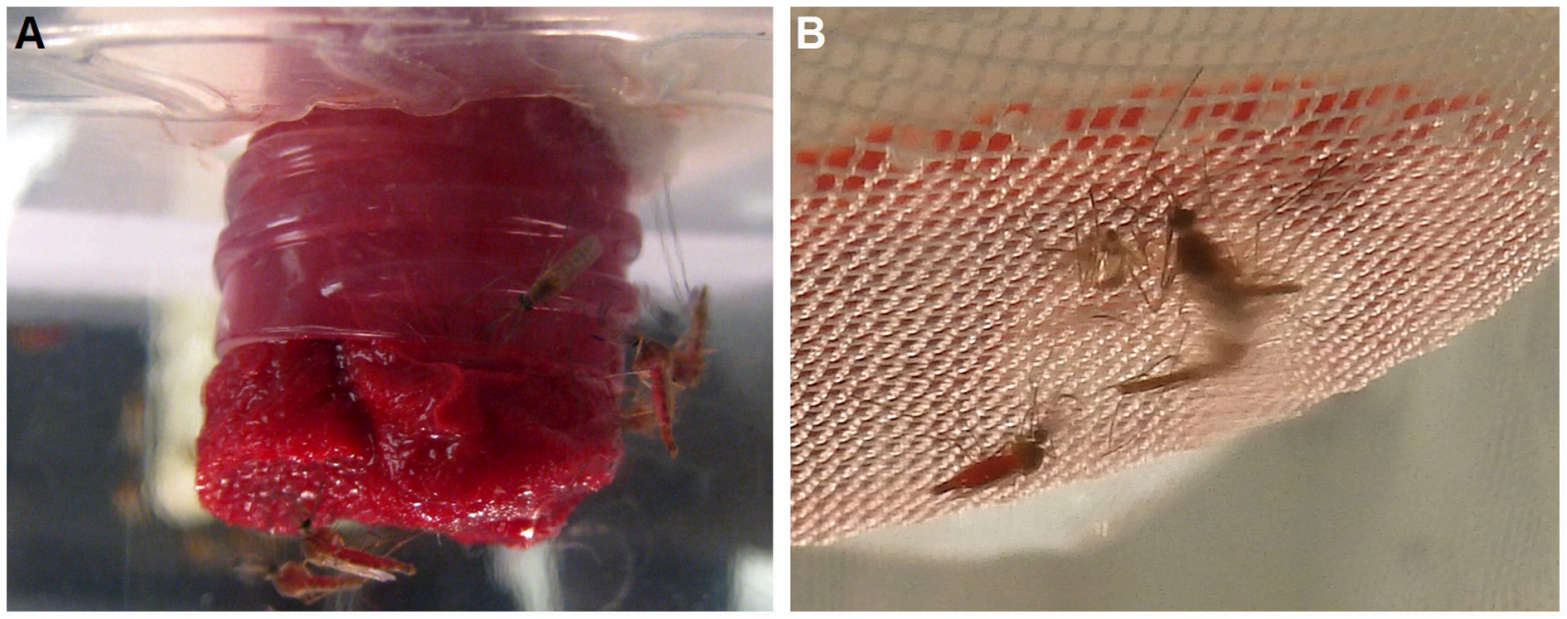
**Blood feeding of male mosquitoes. (A)** Males sitting on upside-down, blood-soaked cotton rolls in a plastic cup. **(B)** Blood feeding of males through a layer of Parafilm®.

To determine male feeding preference (blood vs. sugar), we either used a plastic cup or a rearing cage (Figure S2). If a plastic cup was used, 20 mosquitoes were released into the cup, and two test tubes, containing sugar and blood, were supplied to them (mounted on top, 4.5 cm apart; Figure S2A). For a dual choice cage assay, 20 male specimens were released in the cage and the same sugar and blood tubes were hung at the two opposite corners inside the cage (30 cm apart; Figure S2B). Blood-impregnated cotton rolls used in these assays were not heated, and offered to the males at room temperature. Males were starved for 12 h prior to the experiments. The number of individuals feeding on either of the two choices (Figure 1) was recorded within a time frame of 2 min and analyzed later on (*n* = 8).

### Male blood feeding through a membrane

In order to research whether male mosquitoes are able to penetrate an artificial membrane and get the blood in a manner similar to females, a simple blood feeding apparatus was designed (Figure S3). A 100 ml Schott bottle (Duran, Germany), filled with room temperature water to increase stability, was turned upside down and 0.3 ml blood was poured on the bottom. The blood-filled concave bottom was then covered with a stretched layer of Parafilm (Figure S3A). Our observational cage was in fact a normal rearing cage, whose net was removed and replaced by a transparent food wrap layer at four sides (ClingWrap, Glad Products Co., Oakland, CA, USA). One side of the cage was covered with a sheet of white paper to provide a good background for observation and the top was covered with a piece of white net. Mosquitoes were released into the cage and the blood feeding apparatus was placed on the net (Figure S3B). In each trial, 200 male individuals were released into the cage and their blood feeding behavior was observed for 30 min between 10:30 a.m. and 2:30 p.m. Experiments were recorded by taking photos with an Olympus SP-820UZ digital camera equipped with a super macro lens, and videos with a DCR-DVD 810 Sony Digital camcorder.

### Measuring the volume of an ingested blood meal

Due to the toxicity of conventional cyanohaemoglobin protocols, blood meal volumes were determined using a modified alkaline haematin method, originally derived from Zander et al. (1989). Our adaptation of this method requires that blood is dissolved in a solution of 0.1 N NaOH (Fisher Scientific #: S318-500) and 0.04 M Triton X-100 (Fisher Biotech, CAS # 9002-93-1), hereafter referred to as the alkaline haematin detergent (AHD) reagent. The AHD reagent converts hemoglobin to alkaline haematin D-575, a stable end product with a strong absorbance peak at 575 nm. In this assay, absorbance at 575 nm is proportional to total hemoglobin content between 0 and 7 μl of blood. By this method, unknown blood meal volumes can be estimated by interpolating from a previously generated standard curve. Because of the relatively small blood meal volume from each mosquito, ingested blood volumes were assessed by taking the average blood meal from five mosquito abdomens for each replicate. Either blood fed or non-blood fed mosquito abdomens were dissected under a Zeiss Stemi DV4 stereomicroscope and immediately transferred into a solution of 500 μl AHD reagent in a 1.5 ml microcentrifuge tube (USA Scientific # 1415-9100). For abdomens used in the standard curve, the known volume of blood was added immediately after dissecting the fifth abdomen. After five such abdomens were collected, they were homogenized using a tight fitting microtube pestle (USA Scientific # 1415-5390). In order to estimate blood meal volume, a standard curve was prepared with known volumes of defibrinated sheep blood (0, 1.25, 2.5, 3.75, 5, 7.5, or 10 μl for males and 0, 5, 10, 20, 25, 30, or 35 μl for females) and five non-blood fed male or female abdomens. These volumes gave an average volume of 0, 0.25, 0.5, 0.75, 1, 1.5, or 2 μl and 0, 1, 2, 3, 4, 5, 6, or 7 μl for each male or female mosquito abdomen, respectively. After homogenization, the mixtures were centrifuged at 20,000 × g for 5 min. Four hundred microliters of the supernatant was carefully removed so as not to touch the pellet and was diluted 1:1 in AHD reagent for a final volume of 800 μl. The diluted supernatant was then filtered through a 13 mm 0.2 μM syringe filter (Thermo Fisher Nalgene # 720-1320) to eliminate calculated absorbance due to the light scattering. The filtrate was placed into 1 cm pathlength acrylic cuvettes (Sarstedt # 67.740) and absorbance was measured and recorded at 575 nm. Each known volume on the standard curve was calculated by taking the average of three replicates, each consisting of a known volume of blood homogenized with five mosquito abdomens for both males (*y* = 0.547*x*, *R*^2^ = 0.989) and females (*y* = 0.0448*x*, *R*^2^ = 0.993), respectively. Blood meal determination for blood fed mosquitoes followed the above protocol, with the exception that no blood was added after dissection. Mosquitoes were collected immediately after blood feeding and dissected or placed on ice to prevent blood deterioration. Both male and female blood meal volume measurements were made with five replicates, each with an average of five male or female mosquito abdomens.

### Dissection and measurement of male and female mouth stylets

In order to compare male and female structures, proboscises of both sexes were dissected under a Leica stereo microscope MS5, equipped with an ocular micrometer. Mosquitoes were placed in a glass Petri dish (10 cm diameter), and the mouth parts immersed in a tiny droplet of 1% dimethyl sulfoxide (DMSO) solution to reduce evaporation. Dissection was done with insect pins size 000 (BioQuip) and fine dissecting 5 SA VOMM tweezers (Germany). The length of the proboscis was measured for randomly picked male and female mosquitoes (*n* = 30, for each group). Then, all six piercing stylets were carefully detached and the length of the pair of mandibles and maxillae were measured for males and females (*n* = 10 for each group).

### Statistical analysis and graphical preparations

In order to normalize the distribution of our data from two-choice behavioral assays (herein referred to as groups “a” and “b”), data in each of those two groups were transformed by arc sin √a/(a+b) and arc sin √b/(a+b), respectively, and their percentiles were calculated. This transformation enabled us to use the parametric *t*-test for comparing the behavior of groups “a” and “b.” The distributions of transformed data were verified by a Shapiro-Wilk test for normality at *P* > 0.05, followed by a paired-comparisons *t*-test. Significant differences between several experimental groups were tested by ANOVA, and significantly different groups were recognized by a Waller-Duncan *post-hoc* test. Survival curves were constructed using Kaplan-Meier and analyzed using ANOVA test. All analyses were performed with SPSS statistical package Ver. 16.00 (Chicago, Illinois, USA) and graphics were generated by GraphPad Prism 6 (La Jolla, CA). The moderate number of replicates did not impact the power of statistical analysis.

## Results and discussion

**Figure 2:**
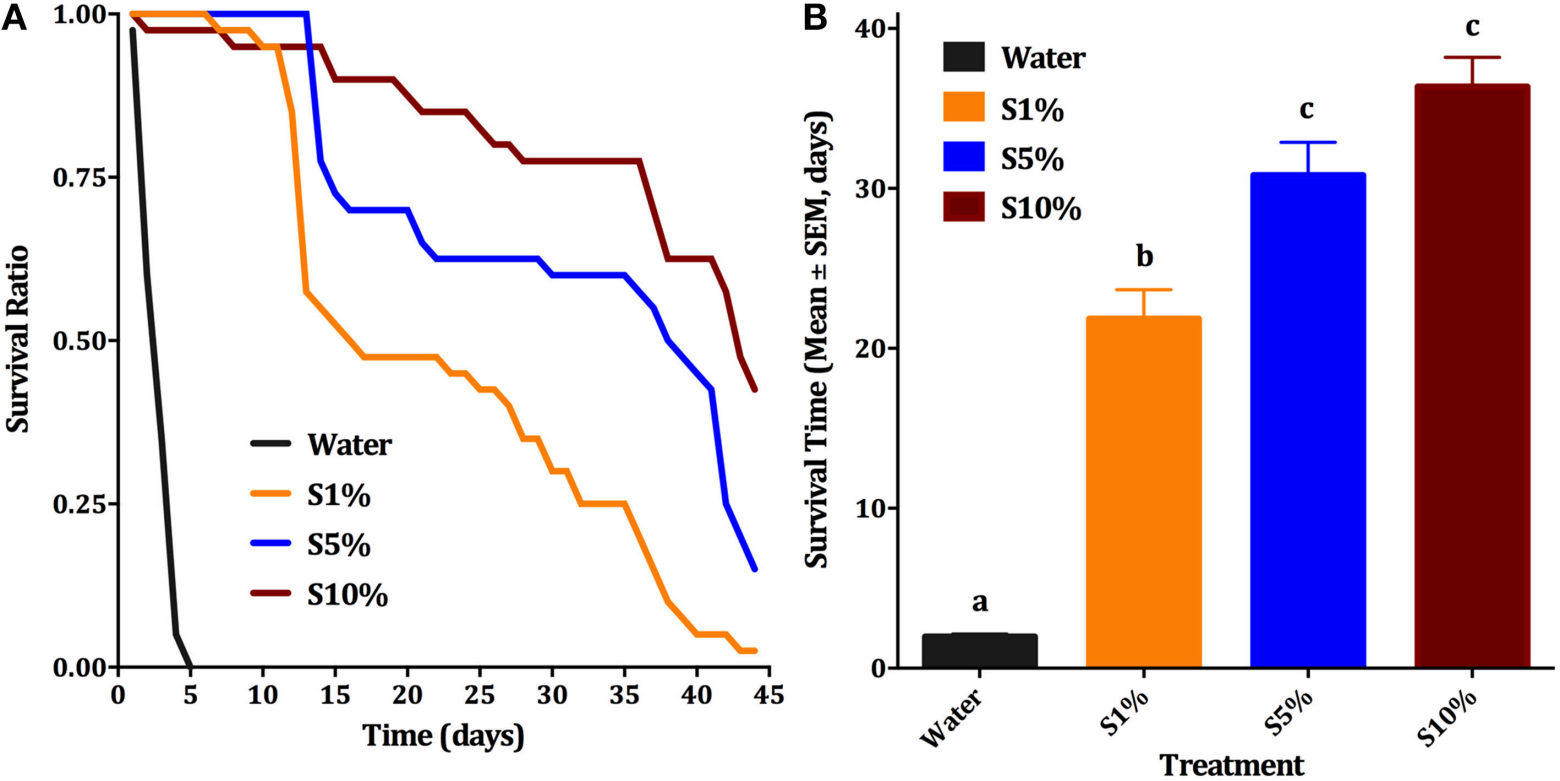
**Effect of sugar concentration on male survival**. **(A)** Survival curves of male *Cx. quinquefasciatus* on: water, 1, 5, and 10% sucrose solutions (S1, S5, and S10%, respectively); Kaplan-Meier test [*n* = 4; Log rank (Mentel-Cox), Chi-square: 84.69]. **(B)** Survival time (Mean days ± SEM) of male *Cx. quinquefasciatus* for the above-described conditions, ANOVA, *F* = 96.314, df = 3, 172, *P* < 0.001, *n* = 4. Waller-Duncan *post-hoc* test. Different letters indicate statistically significant groups.

Blood feeding of male individuals was first observed when females *Cx. quinquefasciatus* were placed along with males in a clear plastic cup for feeding. We noticed that not only females, but also males fed on blood. This feeding easily happened on a blood-soaked cotton roll, hanged in a cup or a cage (typically 20–40% of males fed within 30 min; Figure 1A) and, to a lesser extent, through a layer of Parafilm® (Figure 1B). In marked contrast, for any single membrane feeding observation trial, no more than 5 out of 200 male individuals fed through the membrane. We noticed, however, that blood-fed males did not survive for too long. We then designed a series of experiments to test feeding preference, effect of mating on blood feeding, and effect of blood feeding on male survival. First, we compared male survival on various concentrations of sucrose solutions to provide a baseline for subsequent studies. Survival ratio of males is much higher on all sucrose solutions (over 40 days) than on water (less than 5 days; Figure 2A). Males on 10% sucrose solution had a mean survival time of 36.35 days and along with those on 5% sucrose (30.8 days) had a significantly higher survival time than 1% sucrose (21.8 days) or water (1.9 days; Figure 2B; ANOVA, *F* = 96.314, df = 3, 172, *P* < 0.001, *n* = 4).

**Figure 3:**
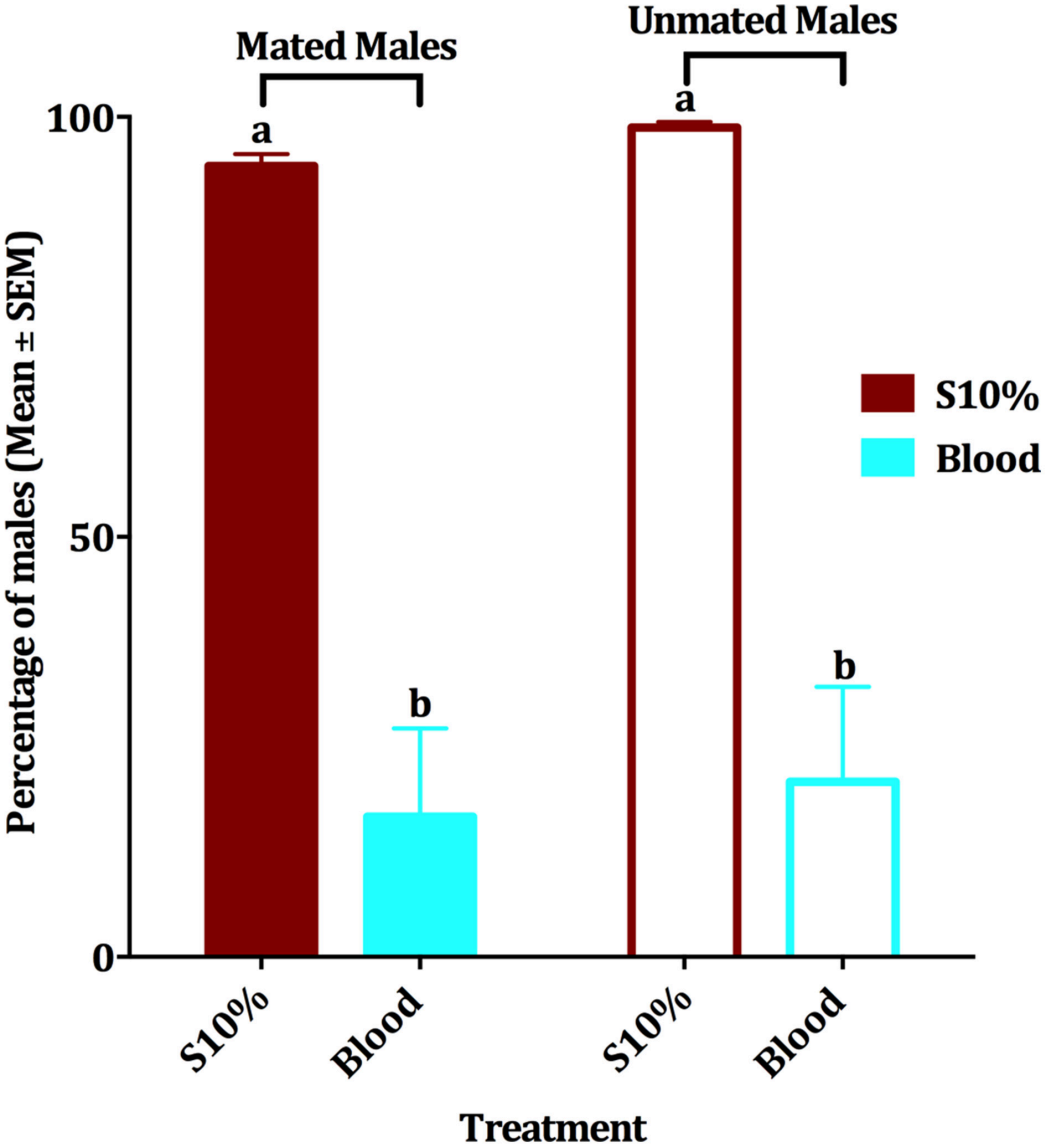
**Effect of blood meal on survival of mated and unmated males**. Mean ± SEM of percentage of mated and unmated survived male *Cx. quinquefasciatus* in a 10-day period experiment. Males had access to either 10% sucrose or blood in a no-choice assay. The four experimental groups were tested by ANOVA, (df = 3, 36, *F* = 33.379, *P* < 0.001, *n* = 4), followed by a Waller-Duncan *post-hoc* test to recognize the significantly different groups, which are denoted by different letters.

Since when we first observed blood feeding by males we did not know the mating status of those individuals, we then compared the effect of blood feeding on survival of mated vs. unmated males. When presented blood and 10% sucrose solution in separate plastic cups, groups of mated and unmated males survived similarly (Figure 3). Mean of survival for the four experimental groups within a period of 10 days was compared by an ANOVA analysis (df = 3, 36, *F* = 33.379, *P* < 0.001, *n* = 4). Since males had been fed with a dyed sucrose solution before being tested with blood, the food dye itself was also tested for possible effect on the male survival. Our experiments showed that the edible food dye had no effect on the mean of male survival in a period of 2 weeks (paired-sample *t*-test, df = 3, *P* = 0.210, *n* = 4 replicates of 10 mosquitoes each; survival >95% after 10 days for both groups). In summary, blood feeding negatively affected survival of mated and unmated males. In the subsequent experiments, we used only mated males.

**Figure 4:**
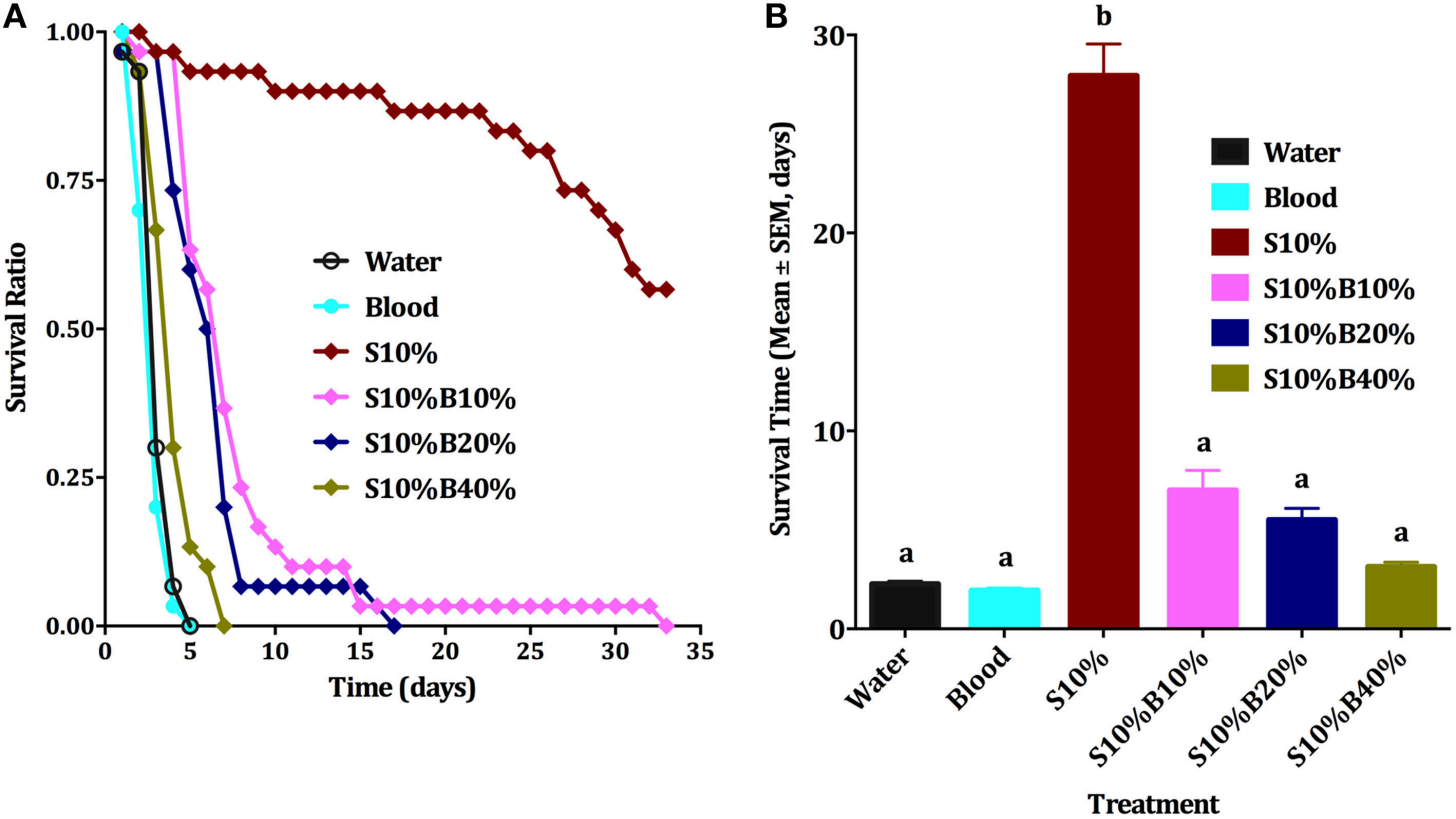
**Male survival on blood vs. sugar. (A)** Survival curve of male *Cx. quinquefasciatus* on: water, 10% sucrose solution (S10%), blood, S10%B10%; S10%B20% and S10%B40%; all blood samples were spiked with 10% sucrose; Kaplan-Meier test [*n* = 3; Log rank (Mentel-Cox), Chi-square: 62.6]. **(B)** Survival time (Mean days ± SEM) of male *Cx. quinquefasciatus* for the six experimental groups described in **(A)**, ANOVA, *F* = 47.564, df = 5, 192, *P* < 0.001, *n* = 3. Waller-Duncan *post-hoc* test. Different letters indicate statistically significant groups.

Although all individuals who fed on blood died very quickly, usually in less than 4 days, the first sets of experiments did not allow us to conclude unambiguously whether blood itself or the lack of sugar (malnutrition) affected survival. Male nutritional reserve right after emergence is usually very low and they cannot survive long without feeding on a source of sugar (Van Handel, 1984; Foster, 1995). The next series of experiments were designed to rule out malnutrition. We compared six groups of male mosquitoes provided with (i) water (negative control), (ii) 10% sucrose (positive control, S10%), (iii) blood only, (iv) 10% sugar, 10% blood (S10%B10%); (v) 10% sugar, 20% blood (S10%B20%), and (vi) 10% sugar, 40% blood (S10%B40%) We observed that males supplied only with water or blood had about the same survival rate (Figures 4A,B) and they could not survive beyond the fifth day, whereas over 58% of males on sugar solution were still alive at day 33. As percentage of blood was increased in blood/sugar mixtures, survival rate of males was reduced (Figure 4B). Means of survival on S10%B10%, S10%B20%, and S10%B40% were 7, 5.5, and 3.1 days, respectively. Survival of males on blood/sugar mixtures did not significantly differ from survival of males on water or blood (Figure 4B). Ten percent sucrose solution was the only treatment on which male mosquitoes survived significantly longer (27.9 days; ANOVA, *F* = 47.564, df = 5, 192, *P* < 0.001, *n* = 3).

Considering that blood is lethal to males, we next asked if males would avoid blood in a two-choice assay. Intriguingly, none of the dual choice experiments in either a small plastic cup (Video S1) or a rearing cage in which blood and sugar solution were simultaneously offered to groups of male mosquitoes revealed any significant difference between the number of males feeding on blood and a 10% sucrose solution within a 2 min time frame. In cage assays with 20 male mosquitoes, there was no significant difference between feeding on sugar (2.87 ± 0.75 males) and on blood (1.75 ± 0.49 males) in a 2-min time frame. Similarly, in cup assays, there was no significant difference between sugar (3.5 ± 0.17 males) and blood (2.87 ± 0.61 males) within 2 min (Paired sample *t*-test, for cup: df = 7, *P* = 0.085, *n* = 8, for cage: df = 7, *P* = 0.195, *n* = 8). The position of 10% sucrose and blood were altered after every replicate to avoid the positioning effect (Video S1). In a separate set of experiments we then compared the amount of blood ingested by male and female mosquitoes in a full blood meal. While the mean male blood meal volume was 0.44 ± 0.02 μl (*n* = 25; five biological replicates of five males each) the mean female blood meal volume was 4.35 ± 0.11 μl (*n* = 25; five biological replicates of five females each). Thus, male acquired in average about 10% of a regular female blood meal. The estimated blood meal volume for female *Cx. quinquefasciatus* is somewhat less than what has been reported in the literature, 5.7 ± 1.8 μl (Knockel et al., 2013). It is conceivable that our method may have underestimated the volume of a mosquito blood meal, in part because we used only the abdomen and, consequently, did not take into account blood retained in other parts of the body.

Lastly, we compared the length of male and female proboscis and observed that female proboscis was slightly longer than that of a male specimen (Paired sample *t*-test, df = 29, *P* = 0.034, *n* = 30). The proboscis is a gutter-shaped tube which encompasses the six piercing stylets in mosquitoes (Choo et al., 2015). We dissected the stylets of the proboscis of both sexes and measured the mandibles and maxillae which, respectively, function as piercing and cutting tools for mosquitoes. Our measurements indicate that, as previously reported (Wahid et al., 2003), maxillae have about the same length in both sexes of *Cx. quinquefasciatus*, but mandibles are significantly shorter in males than in females (Table 1).

**Table 1:** **Mean ± SEM of proboscis, mandible, and maxilla lengths (in mm) in male and female *Cx. quinquefasciatus***.

**Female**			**Male**		
**Proboscis**	**Mandible**	**Maxilla**	**Proboscis**	**Mandible**	**Maxilla**
2.11 ± 0.02^*^	2.08 ± 0.02^a^	2.04 ± 0.01^a^	2.01 ± 0.03	1.53 ± 0.03^b^	2.00 ± 0.02^a^

Data had a normal distribution (Shapiro-Wilk test, P > 0.05). Proboscis length in two sexes were compared by a paired sample t-test, df = 29, t = 2.219, P = 0.034, n = 30; The asterisk shows the significantly longer proboscis. Mandible and maxilla lengths compared by ANOVA, df = 3, 36, F = 95.932, P < 0.001, n = 10, followed by Waller-Duncan post-hoc test; Letters indicate the statistically significant differences among groups.

In blood-sucking females, the toothed maxillae function as hooks and secure the fascicle to the host's skin (Clements, 1992). It is assumed that mandibles' role is in piercing the host's skin as observed in other blood-sucking nematoceran flies, or maybe serve as a valve regulating the size of the distal opening of the labral food canal (Clements, 1992). Maxillae of male mosquitoes have the same length as the female maxillae, but male mandibles are considerably shorter, which is perhaps the reason why male fascicle is not able to suck blood through a human or animal skin. It is, therefore, unlikely that male mosquitoes would be able to reach blood vessels of live animals. If they did, however, they would not survive for too long as blood is toxic to males, which probably are not equipped with enzymes for proper digestion. Indeed, it has been demonstrated that the level of adenosine deaminase (ADA) in females *Aedes aegypti* and *Cx. quinquefasciatus* was over 30 times more than ADA level in males of the same species (Ribeiro et al., 2001) thus suggesting that ADA inactivity in male saliva might be somehow associated with their failure in blood feeding on a host. Additionally, salivary glands of male and female mosquitoes have similar amounts of the enzymes associated with sugar feeding (Marinotti et al., 1990), but males have much less, or none, of the enzyme activities associated with blood feeding, e.g., antiplatelet apyrase enzyme or anticlotting activities (Rossignol et al., 1984; Stark and James, 1995). On the other hand, male mosquitoes have only 5% of the salivary apyrase of their female counterparts, which is needed for blood ingestion in a short time (Rossignol et al., 1984).

It is interesting that despite being toxic, males feed on blood and these findings suggest that males might also have gustatory receptors similar to those in females. Likewise, male ability to detect CO_2_ is also intriguing. Male *Ae. aegypti* house sensilla in maxillary palp, which respond to CO_2_ with sensitivity comparable to that of females. The ability of males to detect CO_2_ had raised questions concerning the potential function of this chemical stimulus in the biology of males (Grant et al., 1995). Our fortuitous discovery that males feed on blood may shed more light on our understanding of CO_2_ reception in male mosquitoes.

## Author contributions

WL conceived the research. WL, MN, and GB designed experiments. MN and GB conducted experiments. MN and GB analyzed data and WL, MN, and GB wrote the manuscript.

## Funding

Research reported in this publication was supported by the National Institute of Allergy and Infectious Diseases of the National Institutes of Health under Award Number R01AI095514. The content is solely the responsibility of the authors and does not necessarily represent the official views of the National Institutes of Health.

### Conflict of interest statement

The authors declare that the research was conducted in the absence of any commercial or financial relationships that could be construed as a potential conflict of interest.

## References

[B1] Anonymous (2014). World Health Statistics 2014. Geneva: World Health Organization.

[B2] Choo Y.-M. Buss G. K. Tan K. Leal W. S. (2015). Multitasking roles of mosquito labrum in oviposition and blood feeding. Front. Physiol. 6:306 10.3389/fphys.2015.00306 PMC4625056 26578978

[B3] Clements A. N. (1992). The Biology of Mosquitoes. London, New York, Wallingford, Oxfordshire, UK; Cambridge, MA: Chapman and Hall; CABI.

[B4] Foster W. A. (1995). Mosquito sugar feeding and reproductive energetics. Annu. Rev. Entomol. 40, 443–474. 10.1146/annurev.en.40.010195.002303 7810991

[B5] Grant A. J. Wigton B. E. Aghajanian J. G. O'Connell R. J. (1995). Electrophysiological responses of receptor neurons in mosquito maxillary palp sensilla to carbon dioxide. J. Comp. Physiol. A 177, 389–396. 10.1007/BF00187475 7674195

[B6] Kelly R. Edman J. D. (1992). Multiple transmission of Plasmodium gallinaceum (Eucoccida: Plasmodiidae) during serial probing by Aedes aegypti (Diptera: Culicidae) on several hosts. J. Med. Entomol. 29, 329–331. 10.1093/jmedent/29.2.329 1495052

[B7] Klowden M. J. (1995). Blood, sex, and the mosquito: the mechanisms that control mosquito blood-feeding behavior. Bioscience 45, 326–331. 10.2307/1312493

[B8] Knockel J. Molina-Cruz A. Fischer E. Muratova O. Haile A. Barillas-Mury C.. (2013). An impossible journey? The development of *Plasmodium falciparum* NF54 in *Culex quinquefasciatus*. PLoS ONE 8:e63387. 10.1371/journal.pone.0063387 23658824 PMC3643899

[B9] Leal W. S. (2014). The enigmatic reception of DEET - the gold standard of insect repellents. Curr. Opin. Insect Sci. 6, 93–98. 10.1016/j.cois.2014.10.007 25530943 PMC4269249

[B10] Marinotti O. James A. Ribeiro J. (1990). Diet and salivation in female *Aedes aegypti* mosquitoes. J. Insect Physiol. 36, 545–548. 10.1016/0022-1910(90)90021-7

[B11] Matheson R. (1944). Handbook of the Mosquitoes of North America; Their Anatomy and Biology; How They Can Be Studied and How Identified; How They Carry Disease and How They Can Be Controlled. Ithaca, NY: Comstock.

[B12] Ribeiro J. M. (1987). Role of saliva in blood-feeding by arthropods. Annu. Rev. Entomol. 32, 463–478. 10.1146/annurev.en.32.010187.002335 2880553

[B13] Ribeiro J. M. Charlab R. Valenzuela J. G. (2001). The salivary adenosine deaminase activity of the mosquitoes Culex quinquefasciatus and *Aedes aegypti*. J. Exp. Biol. 204, 2001–2010. 11441041 10.1242/jeb.204.11.2001

[B14] Rossignol P. A. Ribeiro J. M. Spielman A. (1984). Increased intradermal probing time in sporozoite-infected mosquitoes. Am. J. Trop. Med. Hyg. 33, 17–20. 6696175 10.4269/ajtmh.1984.33.17

[B15] Stark K. R. James A. A. (1995). A factor Xa-directed anticoagulant from the salivary glands of the yellow fever mosquito *Aedes aegypti*. Exp. Parasitol. 81, 321–331. 10.1006/expr.1995.1123 7498429

[B16] Van Handel E. (1984). Metabolism of nutrients in the adult mosquito. Mosq. News 44, 573–579.

[B17] Wahid I. Sunahara T. Mogi M. (2003). Maxillae and mandibles of male mosquitoes and female autogenous mosquitoes (Diptera: Culicidae). J. Med. Entomol. 40, 150–158. 10.1603/0022-2585-40.2.150 12693842

[B18] Zander R. Lang W. Wolf H. U. (1989). The determination of haemoglobin as cyanhaemiglobin or as alkaline haematin D-575. Comparison of method-related errors. J. Clin. Chem. Clin. Biochem. 27, 185–189. 10.1515/cclm.1989.27.4.185 2472459

