# Supplementary material for: Toxic Effect of Blood Feeding in Male Mosquitoes
Source: Front Physiol. 2016 Jan 26;7:4. doi: 10.3389/fphys.2016.00004 (PMC4726748; doi:10.3389/fphys.2016.00004)
Supplement: Supplementary file 2 [file DataSheet1.DOCX]

**Supplementary Material**

Hematophagic male mosquitoes

Nikbakhtzadeh et al.

**Supplementary Figure Legends**

**FIGURE S1. Modified cup used in no-choice feeding assays.** Side view of the cup with a15 ml centrifuge tube placed upside down through an opening on the lid. The tube was filled with water, sugar (as shown), blood, or a mixture of blood and sugar.

**FIGURE S2. Dual-choice feeding arenas. (A)** Bird’s-eye view of the cup dual-choice arena pictured with tubes filled with sugar on the left and blood on the right, and a camera setup on the bottom. **(B)** Perspective view of the cage dual-choice arena showing test tubes filled with blood on the left and sugar on the right.

**FIGURE S3. Membrane-feeding observation cage. (A)** 100 ml Schott bottle, filled with water, and placed upside down. Blood is containing between the concave base of the bottle and a layer of Parafilm™ stretched over the top. **(B)** The previously described membrane feeding apparatus, placed on the top of the observation case with the membrane in direct contact with the mesh cover.

**Supplementary Video Legend**

**Video S1. Real-time videos of two-choice feeding assays.** Three 1-minute-video-clips from multiple dual-choice feeding assays showing blood and 10% sugar at different sides of the arena. Bioassay 3 was performed with 40 males for illustration only. Detailed male feeding behavior, particularly insertion and subsequent cleaning of the proboscis, is highlighted in the last 30 sec segment of the video.
