# Supplementary figures and images for: Toxic Effect of Blood Feeding in Male Mosquitoes
Source: Front Physiol. 2016 Jan 26;7:4. doi: 10.3389/fphys.2016.00004 (PMC4726748; doi:10.3389/fphys.2016.00004)

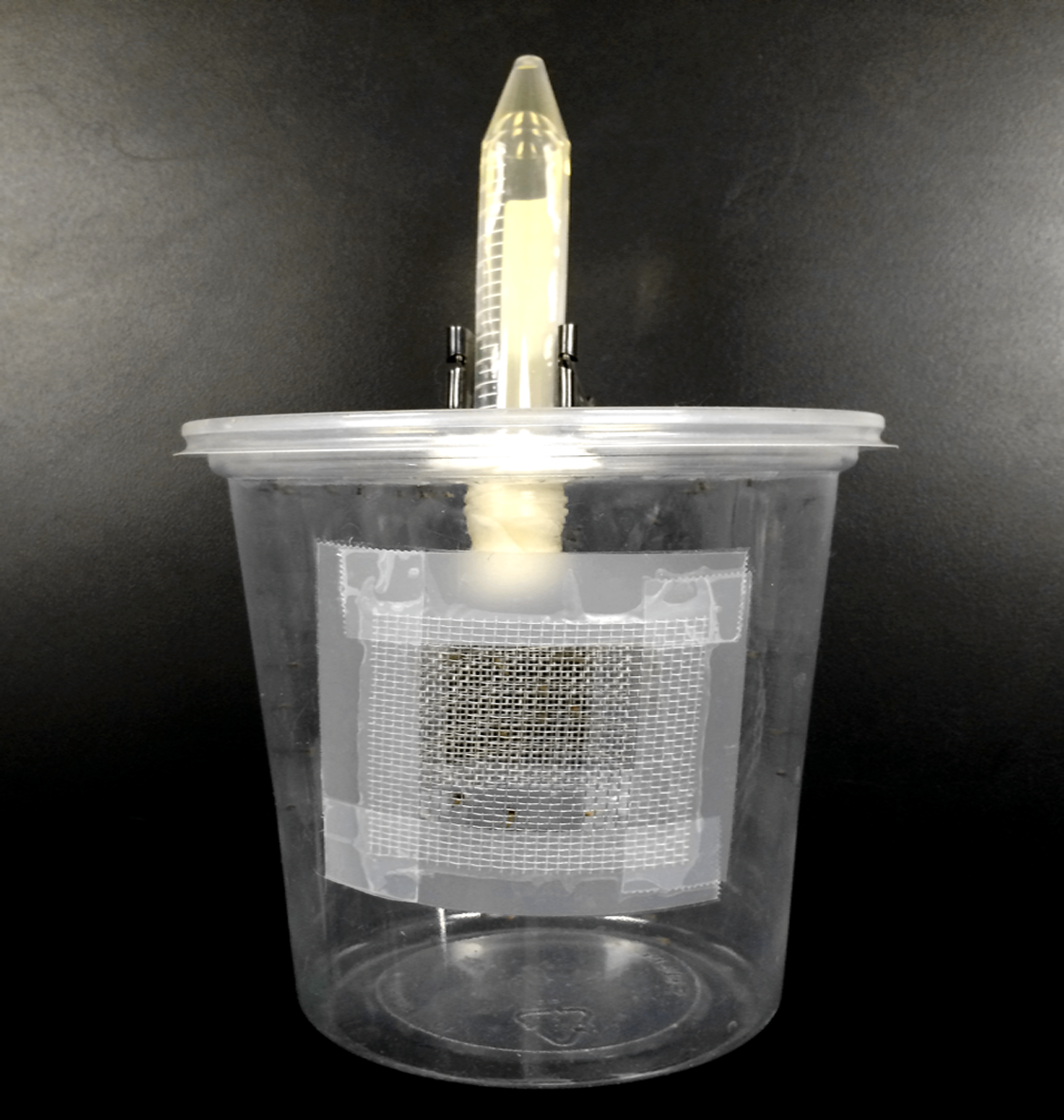

Supplement: Supplementary file 3 [file Image1.TIFF]

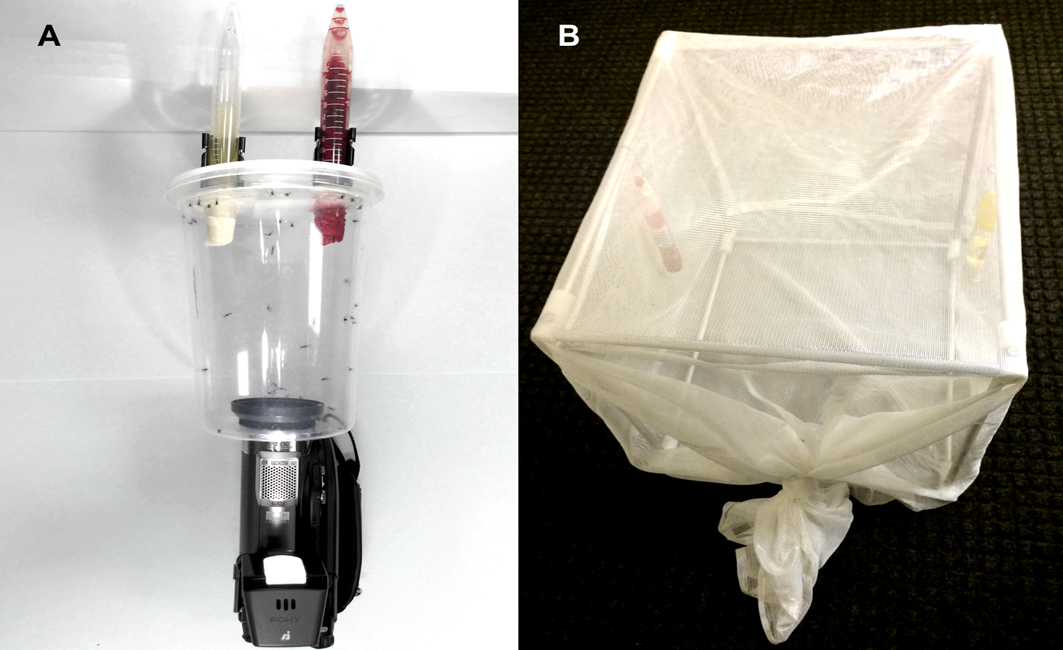

Supplement: Supplementary file 4 [file Image2.TIFF]

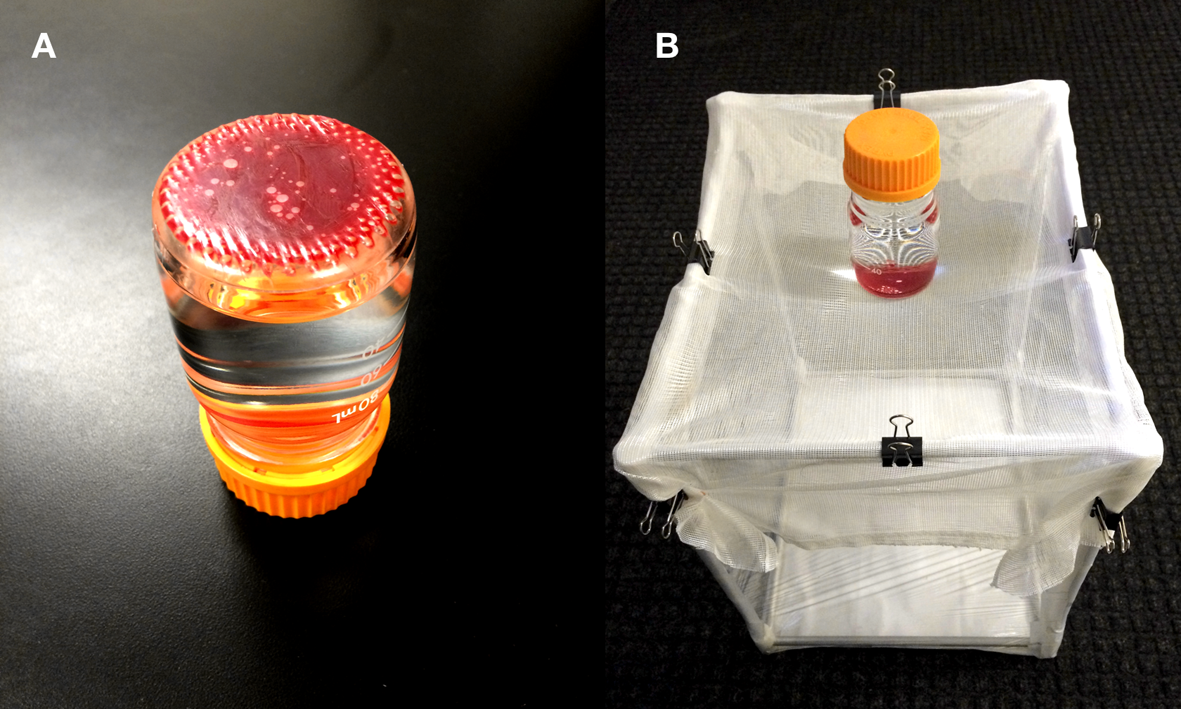

Supplement: Supplementary file 5 [file Image3.TIFF]
